# Preservation of Beauty

**Published:** 1893-08

**Authors:** John J. Lewis


### PRESERVATION OF BEAUTY.

The rules which I would lay down for the preservation of the bloom of beauty, during its natural life, are few, and easy of access. And besides, having the advantage of speaking of my own wide and minute observations, I have the authority of the most eminent physicians of every age, to support my argument.

The secret of preserving beauty lies in three things— *Temperance*, exercise and cleanliness. From these three heads I hope much good instruction may be deduced. *Temperance* includes moderation at table, and in the enjoyment of what the world calls pleasure. A young beauty, were she fair as Hebe, and elegant as the goddess of love herself, would soon lose these charms by a course of inordinate eating, drinking and late hours.

I think that my delicate young readers will start at this last sentence, and wonder how it can be that any well-bred woman should think it possible that pretty ladies could be guilty of either of the two first mentioned excesses. But, when I speak of *inordinate* eating and drinking, I do not mean feasting like a glutton, or drinking to intoxication. My objection is not more against the *quantity* than the *quality* of the dishes which constitute the usual repasts of women of fashion.

Their breakfasts not only set forth tea and coffee, but chocolate, and *hot* bread and butter.

Both of these latter articles, when taken constantly, are hostile to health and female delicacy. The heated grease, which is their principal ingredients, deranges the stomach ; and by creating or increasing bilious disorders, gradually overspreads the fair skin with a wan or yellow hue. After this meal, a long and exhausting fast not unfrequently succeeds, from ten in the morning till six or seven in the evening, when dinner is served up ; and the half-famished beauty sits down to sate a keen appetite with rich soups, fish, roast and boiled meat, tarts, sweetmeats, ices, etc.

How must the constitution suffer under the digestion of this *melange !* How does the heated complexion bear witness to the combustion within !

And when we consider that the beverage she takes to dilute this mass of food and assuage the consequent fever in her stomach, is not only water from the spring, but champagne, madeira, and other wines, foreign and domestic ; you cannot wonder that I should warn the inexperienced creature against intemperance. The superabundance of aliment which she takes in at this time, is not only destructive of beauty, but the period of such depletion is full of other dangers.

Long fasting wastes the powers of digestion, and weakens the springs of life. In this enfeebled state, at the hour when nature intends we should prepare for general repose, we put our stomachs and animal spirits to extraordinary exertion. Our vital functions are overtaxed and overloaded—we become hectic—for observation strongly declares that invalids and delicate persons should rarely eat solids after three o'clock in the day, as fever is generally the consequence, and thus, almost every complaint that distresses and destroys the human frame may be engendered.

Besides, when we add to this evil the present mode of bracing the digestive part of the body in what is called stays or lacing, to what an extent must reach the baneful effects of a protracted and abundant repast.

Indeed, I am fully persuaded that long fasting, late dining, and the excessive repletion then taken into the exhausted stomach, and the midnight, nay, morning hours, of lingering pleasure, are the positive causes of colds taken, bilious fevers, consumption and atrophies.

By the means enumerated, the firm texture of the constitution is broken, and the principles of health being in a manner decomposed, the finest parts fly off, and the dregs maintain the poor survivor of herself in a sad kind of artificial existence. Delicate proportion gives place either to miserable leanness or shapeless fat. The once fair skin assumes a pallid rigidity, or a bloated redness, which the vain possessor would still regard as the roses of health and beauty.

To repair these ravages comes the aid of padding, to give shape where there is none; stays to compress into form the chaos of flesh; and paints of all hues to rectify the disorder of the complexion.

But useless are these attempts. If dissipation, disease and immoderation have wrecked the fair vessel of female charms, it is not in the power of Esculapius himself to refit the scattered bark; or of Syrens, with all their songs and wiles, to conjure its battered sides from the rocks, and make it ride the sea in gallant trim again.

Let us turn from the ruin of all that is beauteous and lovely, to the cheering hope of preserving every charm unimpaired; and by means which the most ingenuous mind need not blush to acknowledge.

The rules are few. First, *Temperance*: A well timed use of the table, and so moderate a pursuit, that the midnight ball, assembly and theatre shall not too frequently recur.

The next specific is that of daily exercise in the open air. This may be almost always attained, either on horseback, by cycle, or on foot in fine weather; and when that is denied, in carriage.

The morning, two hours after sunrise, is the most salubrious time for a walk or ride. In short, the morning and evening dew, and the unrepelled blaze of a summer noon, must alike be ever avoided as the enemies of health and beauty.

Cleanliness is of most powerful efficacy. It maintains the limbs in their pliancy, the skin in its softness, the complexion in its lustre, the eyes in their brightness, the teeth in their purity, and the constitution in its fairest vigor. To promote cleanliness nothing is preferable to bathing.

The frequent use of tepid (warm) baths is not more grateful to the sense than it is salutary to the health, and to beauty. As ours is a climate subject to sudden heats, colds and rains, tepid immersion is the *only* sovereign remedy against their usual morbific effects. Every lady should make a bath as indispensable an article in her house as a looking-glass.

JOHN J. LEWIS, M. D.

